# Metabotropic glutamate receptor 5 contributes to inflammatory tongue pain via extracellular signal-regulated kinase signaling in the trigeminal spinal subnucleus caudalis and upper cervical spinal cord

**DOI:** 10.1186/1742-2094-9-258

**Published:** 2012-11-27

**Authors:** Ming-Gang Liu, Shingo Matsuura, Masamichi Shinoda, Kuniya Honda, Ikuko Suzuki, Kazuo Shibuta, Takaaki Tamagawa, Ayano Katagiri, Masaaki Kiyomoto, Kinuyo Ohara, Akihiko Furukawa, Kentaro Urata, Koichi Iwata

**Affiliations:** 1 Department of Physiology, Nihon University School of Dentistry, 1-8-13 Kandasurugadai, Chiyoda-ku, Tokyo 101-8310, Japan; 2 Department of Brain and Cognitive Sciences, College of Natural Sciences, Seoul National University, Seoul, 151-746, Korea; 3 Department of Endodontics, Nihon University School of Dentistry, 1-8-13 Kandasurugadai, Chiyoda-ku, Tokyo, 101-8310, Japan; 4 Division of Functional Morphology, Dental Research Center, Nihon University School of Dentistry, Tokyo 101-8310, Japan; 5 Department of Oral and Maxillofacial Surgery, Nihon University School of Dentistry, 1-8-13 Kandasurugadai, Chiyoda-ku, Tokyo, 101-8310, Japan; 6 Department of Psychosomatic Dentistry, Tokyo Medical and Dental University Graduate School, Tokyo, Japan; 7 Department of Oral Diagnosis, Nihon University School of Dentistry, 1-8-13 Kandasurugadai, Chiyoda-ku, Tokyo, 101-8310, Japan; 8 Department of Complete Denture Prosthodontics, Nihon University School of Dentistry, 1-8-13 Kanda-surugadai, Chiyoda-ku, Tokyo, 101-8310, Japan; 9 Division of Applied System Neuroscience Advanced Medical Research Center, Nihon University Graduate School of Medical Science, 30-1 Ohyaguchi-Kamimachi Itabashi, Tokyo, 173-8610, Japan

**Keywords:** Metabotropic glutamate receptor 5, Extracellular signal-regulated kinase, Tongue pain, Inflammation, Trigeminal subnucleus caudalis, Upper cervical spinal cord

## Abstract

**Background:**

In the orofacial region, limited information is available concerning pathological tongue pain, such as inflammatory pain or neuropathic pain occurring in the tongue. Here, we tried for the first time to establish a novel animal model of inflammatory tongue pain in rats and to investigate the roles of metabotropic glutamate receptor 5 (mGluR5)-extracellular signal-regulated kinase (ERK) signaling in this process.

**Methods:**

Complete Freund’s adjuvant (CFA) was submucosally injected into the tongue to induce the inflammatory pain phenotype that was confirmed by behavioral testing. Expression of phosphorylated ERK (pERK) and mGluR5 in the trigeminal subnucleus caudalis (Vc) and upper cervical spinal cord (C1-C2) were detected with immunohistochemical staining and Western blotting. pERK inhibitor, a selective mGluR5 antagonist or agonist was continuously administered for 7 days via an intrathecal (i.t.) route. Local inflammatory responses were verified by tongue histology.

**Results:**

Submucosal injection of CFA into the tongue produced a long-lasting mechanical allodynia and heat hyperalgesia at the inflamed site, concomitant with an increase in the pERK immunoreactivity in the Vc and C1-C2. The distribution of pERK-IR cells was laminar specific, ipsilaterally dominant, somatotopically relevant, and rostrocaudally restricted. Western blot analysis also showed an enhanced activation of ERK in the Vc and C1-C2 following CFA injection. Continuous i.t. administration of the pERK inhibitor and a selective mGluR5 antagonist significantly depressed the mechanical allodynia and heat hyperalgesia in the CFA-injected tongue. In addition, the number of pERK-IR cells in ipsilateral Vc and C1-C2 was also decreased by both drugs. Moreover, continuous i.t. administration of a selective mGluR5 agonist induced mechanical allodynia in naive rats.

**Conclusions:**

The present study constructed a new animal model of inflammatory tongue pain in rodents, and demonstrated pivotal roles of the mGluR5-pERK signaling in the development of mechanical and heat hypersensitivity that evolved in the inflamed tongue. This tongue-inflamed model might be useful for future studies to further elucidate molecular and cellular mechanisms of pathological tongue pain such as burning mouth syndrome.

## Background

There are marked differences in pathological pain following tissue injury or inflammation between deep and superficial tissues [1-3]. Orofacial deep tissue (such as temporomandibular joint or masseter muscle) inflammation produces a stronger excitation and/or sensitization in the trigeminal nervous system compared to cutaneous (such as perioral and facial skin) inflammation [4-6]. Additionally, there are site-related differences in pain processing between intraoral mucosa and facial skin [7,8]. Indeed, these two kinds of orofacial pain have been reported to differ in either the morphology of primary afferent neurons [9] or the somatotopy of trigeminal projections in the brainstem [10]. However, few researchers have examined the differences in mechanisms underlying intraoral mucosal inflammatory pain compared with face inflammatory pain [8,11,12].

Extracellular signal-regulated kinase (ERK), a member of the mitogen-activated protein kinase (MAPK) family, has been demonstrated to play a role in nociceptive transmission, modulation and integration in neurons [13,14]. ERK is phosphorylated following various noxious stimuli, and phosphorylated ERK (pERK) affects the neuronal excitability and pain processing [15-18]. In the orofacial pain system, we have demonstrated that rostrocaudal distribution of pERK-immunoreactive (IR) cells is more extensive in the trigeminal spinal subnucleus caudalis (Vc) and upper cervical spinal cord (C1-C2) after capsaicin injection into the intraoral mucosa than facial skin [12]. Using many different pain models, we further show that pERK-IR neurons in the Vc and C1-C2 play an important role in orofacial inflammatory or neuropathic pain [11,19-24]. Hence, ERK phosphorylation is among the possible mechanisms underlying pathological pain following peripheral inflammation or nerve injury. Moreover, ERK was phosphorylated in Vc neurons within 5 to 10 min following noxious stimulation of the facial skin and the number of pERK-IR cells increased following increases in the noxious stimulus intensity, indicating that ERK phosphorylation in Vc neurons could be used as an excitable marker of nociceptive neurons [20].

Subsequent to activation of primary sensory neurons, glutamate is released from primary afferent terminals in the spinal dorsal horn, and then ionotropic ligand-gated glutamate receptors and G-protein-coupled metabotropic glutamate receptors (mGluRs) are activated [25]. mGluR5, a subgroup of the mGluRs, is reported to be highly expressed and thus involved in nociceptive processing in the spinal dorsal horn [26-28]. Intrathecal (i.t.) administration of the mGluR1/5 agonist induced spontaneous nocifensive behavior as well as thermal hyperalgesia and allodynia in rats [29,30]. I.t. pretreatment with the mGluR5 antagonist relieved inflammatory hyperalgesia induced by complete Freund's adjuvant (CFA) injection into the hind paw [26]. In addition, peripherally located mGluR5 has been shown to participate in the generation of mechanical allodynia in either vibrissa pad [31] or masseter muscle [32], while centrally located mGluR5 is reported to contribute to the induction of long-term potentiation of primary afferent synaptic transmission in the Vc of juvenile rats [33]. Interestingly, evidence has also been provided to reveal that mGluR5 modulates inflammatory nociceptive plasticity via downstream activation of ERK signaling in the spinal cord [34-36].

Taken together, the aims of the present study are two-fold. First is to establish a novel experimental model of inflammatory tongue pain by submucosal injection of CFA into the tongue. Second, we sought to test the hypothesis that activation of mGluR5 is important for ERK phosphorylation in Vc and C1-C2 neurons after tongue inflammation and that this pathway is necessary for the hyperexcitability of Vc and C1-C2 neurons following CFA injection into the tongue, resulting in an enhancement of nocifensive behaviors to heat and mechanical stimuli.

## Methods

### Animals

Adult male Sprague Dawley rats (200 to 250 g) were used in this study (Japan SLC, Japan). Rats were exposed to a 12 h light/dark cycle and kept in a temperature-controlled room (23 ± 2°C) with food and water *ad libitum*. This study was approved by the Animal Experimentation Committee at Nihon University, and all experimental procedures were performed according to the ethical guidelines of the International Association for the Study of Pain [37]. All possible efforts were made to minimize the number of animals used and their suffering.

### Drugs

The major drugs used in the current study were PD98059 (EMD Biosciences, La Jolla, CA, USA), a well-known MAPK kinase (MEK1/2) inhibitor [38], 2-methyl-6-(phenylethynyl)-pyridine (MPEP, Sigma Aldrich, St. Louis, MO, USA), a selective mGluR5 antagonist [39], (RS)-2-Chloro-5-hydroxyphenylglycine (CHPG, Tocris, Bristol, UK), a selective mGluR5 agonist [40] and CFA (an inflammatory agent, Sigma Aldrich). PD98059 and MPEP were initially dissolved in 100% dimethyl sulfoxide (DMSO) as stock solutions (10 μg/μL) for frozen aliquots, and then further diluted to 0.1 μg/μL in 10% DMSO (in 0.9% saline) for i.t. administration (see below). A solution of 10% DMSO served as the vehicle control. CHPG was diluted to 4.8 mM in 0.9% saline for i.t. administration. For preparation of CFA solution, the original drug was suspended in an oil/saline (1:1) emulsion and stored at 4°C for subsequent use.

### Induction and verification of inflammation in the tongue

Under anesthesia from an intraperitoneal injection of sodium pentobarbital (50 mg/kg, Schering Plough, Whitehouse Station, NJ, USA), 5 μL of CFA was submucosally injected into the left side of the anterior dorsolateral two-thirds of the tongue with a 30-gauge needle attaching a Hamilton syringe (1.0 mL, Hamilton, Reno, NV, USA). The same amount of isotonic saline was injected as the vehicle control. Notably, during each injection, the location of the needle was best limited in the superficial skin layer of the tongue without entering the tongue muscle. After the injection, a small cotton swab was placed on the injection site for 1 to 2 min to prevent any leakage. Animals were closely monitored for evidence of distress or pain, and weight gain following the injections.

To verify the occurrence of tongue inflammation, rats were perfused transcardially with 250 mL 0.9% isotonic saline followed by 500 mL ice cold 4% paraformaldehyde in 0.1 M phosphate buffer (PB, pH 7.4) on days 8 and 15 after CFA injection. The tongues were removed and immersed in the same fixative for 4 h at 4°C. After post-fixation, tongue tissues were embedded in Tissue Tek (Sakura Finetechnical, Tokyo, Japan), cut in the horizontal plane along the long axis of the tongue on a cryostat at a thickness of 10 μm, stained with hematoxylin and eosin (HE), and evaluated microscopically.

To examine inflammatory extravasation at each specified time point, Evans Blue solution (50 mg/kg, 10 mg/mL in saline) was intravenously injected through the femoral vein at 4 to 5 min before perfusion. Then rats were perfused through the aorta with normal saline. Photographs of tongue sections were taken, and Evans Blue stained regions were observed under the microscope.

### Behavioral testing

Rats were lightly anesthetized with 2% isoflurane (Mylan, Morgantown, WV, USA) mixed with oxygen, and the depth of anesthesia was assured as previously described [24]. According to our previous reports [11,20,41,42], a pair of bipolar enamel-coated silver-wire electrodes was inserted into the splenius capitis muscle for electromyographic (EMG) recording (inter-electrode distance, 5 to 6 mm). The EMG activity was amplified, filtered, digitized, and integrated by the Spike 2 software (CED 1401, Cambridge Electronic Design, Cambridge, UK).

For measurement of mechanical head withdrawal threshold (MHWT), an electronic von Frey anesthesiometer (Bioseb, Chaville, France) was used to apply graded mechanical pinch stimuli to CFA- or saline-injected tongue. The MHWT was defined as the lowest pressure (g) required to elicit a robust bursting activity in neck EMG recording accompanied by a clear head withdrawal response. The cutoff mechanical stimulus intensity was 130 g. For assessment of heat head withdrawal threshold (HHWT), heat stimulus was applied using a contact thermal probe (25 mm^2^, adapted temperature: 35°C, Intercross, Tokyo, Japan) to the tongue. The probe temperature increased 0.3°C per second during the assessment period. The HHWT was defined as the minimum temperature sufficient to elicit a drastic head escape and sudden appearance of a bursting EMG activity. The cutoff temperature was set at 60°C.

Both MHWT and HHWT were measured 1 day before and on days 1, 3, 5, 8, 11, and 15 after saline or CFA injection. Three measurements (at 5-min intervals) were performed and averaged at each time point for each animal. All behavioral tests were conducted under blind conditions.

### Tissue preparation and pERK immunohistochemistry

On days 3, 8, and 15 after saline or CFA injection into the tongue and in naive rats, noxious mechanical stimulation was applied to the tongue using an arterial clip (intensity, 120 g; duration, 30s; interval, 30s; total, 10 min) with the rats under sodium pentobarbital anesthesia (50 mg/kg, i.p.). On the basis of our previous results that the number of pERK-IR cells peaked at 5 min after capsaicin injection into the tongue [12], rats were perfused transcardially with 250 mL isotonic saline followed by 500 mL cold 4% paraformaldehyde in 0.1 M PB (pH 7.4) at 5 min after noxious stimulation. Furthermore, naive and CFA-injected rats were perfused transcardially in the absence of noxious mechanical stimulation. The medulla and upper cervical spinal cord were removed and placed in the same fixative overnight at 4°C. These tissues were transferred to 20% sucrose in 0.01 M phosphate-buffered saline (PBS) for several days for cryoprotection. Thirty micrometer thick sections of the medulla and upper cervical spinal cord were cut with a freezing microtome at -20°C, and every fourth section was collected in 0.01 M PBS. Free-floating sections were rinsed in 0.01 M PBS, blocked in 3% normal goat serum (NGS) for 1 h at room temperature (RT), and then incubated with rabbit anti-pERK (Thr202/Tyr204) antibody (1:1,000, Cell Signaling Technology, Beverly, MA, USA) in 3% NGS with 0.75% Triton X-100 for 72 h at 4°C. After rinsing, sections were incubated with biotinylated goat anti-rabbit antibody (1:600, Vector Laboratories, Burlingame, CA, USA) for 2 h at RT. Following rinses in 0.01 M PBS, these sections were reacted with peroxidase-conjugated avidin-biotin complex (ABC, 1:50, Vector Laboratories) for 1 h at RT. They were washed in 0.05 M Tris buffer (TB) and incubated with 0.035% 3,3’-diaminobenzidine-tetra hydrochloride (DAB, Tokyo Chemical Industry, Tokyo, Japan) in 0.05 M TB containing 0.2% nickel ammonium sulfate and 0.05% hydrogen peroxide for 3 to 4 min. Sections were finally rinsed in 0.01 M PBS, mounted on gelatin-coated slides, air-dried, dehydrated in ethanol, cleared in xylene, and coverslipped. All specific staining was abolished by omission of the primary antibody in the process.

The pERK-IR cells were counted under a light microscope with an attached camera lucida drawing tube (Neurolucida 2000, MicroBrightField, Colchester, UT, USA). The sections were then grouped into 720 μm segments rostrocaudally with reference to the obex. In order to analyze rostrocaudal distribution of pERK-IR cells, the number of pERK-IR cells from three sections at each level in Vc and C1-C2 was counted, and then averaged from five rats in each group. The cells showing more intense staining than the average background were considered positive for pERK immunoreactivity. The whole counting process was performed by an investigator blind to the experimental treatments.

### Double immunofluorescence

Three rats received noxious mechanical stimulation of the tongue by an arterial clip (intensity, 120 g; duration, 30s; interval, 30s; total, 10 min) on day 8 after CFA injection into the tongue. Five min after the stimulation, rats were perfused with 250 mL isotonic saline followed by 500 mL cold 4% paraformaldehyde in 0.1 M PB (pH 7.4). After cryoprotection in 20% sucrose, 30 μm thick sections were cut as described previously and processed for double immunofluorescence labeling between pERK or mGluR5 and the neuronal label, NeuN, or the astroglial label, glial fibrillary acidic protein (GFAP). We also performed double-immunostaining between pERK and mGluR5 antibodies. Free-floating tissue sections were rinsed in 0.01 M PBS, blocked in 3% NGS for 1 h and incubated with rabbit anti-pERK antibody (1:300, Cell Signaling Technology) or goat anti-mGluR5 antibody (1:50, Santa Cruz, CA, USA) and mouse anti-NeuN antibody (1:1,000, Chemicon, MA, USA), or mouse anti-GFAP antibody (1:1000, Dako, Tokyo, Japan) for 72 h at 4°C. Similarly, free-floating tissue sections were incubated with rabbit anti-pERK antibody and goat anti-mGluR5 antibody (Merck Millipore, Billerica, MA, USA). After rinsing in 0.01 M PBS, the sections were incubated with the secondary antibodies (anti-rabbit or anti-goat Alexa Fluor 488 and anti-rabbit or anti-mouse Alexa Fluor 568, 1:1,000, Invitrogen, NY, USA) where appropriate for 2 h at RT in a dark room. Then, the sections were rinsed in 0.01 M PBS, mounted on slides, and coverslipped with PermaFluor (Sigma Aldrich, St. Louis, MO, USA). The immunofluorescence images were taken with a confocal laser scanning microscope (LSM 510 V3.2, Carl Zeiss, Tokyo, Japan).

### Western blotting

On day 8 after CFA or saline injection into the tongue, the rat was anesthetized with sodium pentobarbital (50 mg/kg, i.p.) and perfused with saline. Medulla containing Vc and C1-C2 was taken out and homogenized in 100 μL of ice-cold lysis buffer (137 mM NaCl, 20 mM Tris-HCl, pH 8.0, 1% NP40, 10% glycerol, 1 mM phenylmethylsulfonyl fluoride, 10 μg/mL aprotinin, 1 g/mL leupeptin, and 0.5 mM sodium vanadate) using a tube pestle (Thermo Fisher Scientific, Waltham, MA, USA). Sample was centrifuged at 15,000 rpm for 10 min at 4°C. The supernatant was collected to new tubes and protein concentration of the sample was determined with a protein assay kit (Bio-Rad, CA, USA). Protein sample was heat-denatured in Laemmli sample buffer solution (Bio-Rad). Sample (30 μg) was subjected to electrophoresis for protein separation on 10% SDS-PAGE and electroblotted onto polyvinylidene fluoride membranes (Trans-Blot turb Transfer pack, Bio-Rad) by using Trans-Blot Turbo (Bio-Rad). Following rinsing with Tris-buffered saline containing 0.1% Tween 20 (TBST), the membrane was incubated with 3% bovine serum albumin (BSA, Bovogen, Essendon, Australia). The membrane was incubated overnight at 4°C with anti-pERK antibody (1:1,000, Cell Signaling) or anti-mGluR5 antibody (1:20,000, Merck Millipore) diluted in TBST containing 5% BSA. Each protein binding was visualized using a horseradish peroxidase-conjugated donkey anti-rabbit antibody (Cell signaling) and Western Lightning ELC Pro (Perikin Elmer, Waltham, MA, USA). Band intensity was quantified using a ChemiDoc MP system (Bio-Rad) and normalized to β-actin immunoreactivity on blots reprobed with anti-β-actin antibody (1:200, Santa Cruz) after removing protein binding using a stripping reagent (Thermo Fisher Scientific).

### Effects of PD98059, MPEP and CHPG on nocifensive behavior and ERK phosphorylation

Rats were anesthetized with sodium pentobarbital (50 mg/kg, i.p.) and placed in a stereotaxic apparatus. After a midline skin incision, an opening was made in the caudal part of the skull with a dental drill to insert intrathecally a soft polyethylene tube (PE45, ID, 0.58 mm; OD, 0.96 mm; Natsume, Tokyo, Japan) [43]. The tube was connected to a mini-osmotic pump (Alzet model 2001, Alzet, Cupertino, CA, USA; total volume, 200 μL) filled with the drug and embedded subcutaneously in the dorsal portion of the body. Thus, PD98059 or MPEP was intrathecally applied for 7 days (1.0 μL/h). The dosage and duration of the two drugs (0.1 μg/μL) for pump infusion were chosen primarily based on previous reports [11,20,23,39]. After completion of the pump embedding, 5 μL of CFA solution was injected into the anterior dorsolateral two-thirds of the rat tongue, and the behavioral testing was performed on days 1, 3, 5, 8, 11, and 15 for PD98059 treatment or on day 8 for MPEP treatment after CFA injection. CHPG was also intrathecally applied for 7 days (0.5 μL/h) in naive rats and behavioral testing was performed on day 8.

On day 8, CFA-treated rats with continuous PD98059 or MPEP administration received noxious mechanical stimulation to the tongue. Five min after the stimulation, the rats were perfused, and the pERK immunohistochemistry was performed. All subsequent staining steps and counting analysis were the same as described above. All behavioral and immunohistochemical experiments were conducted on animals without any obvious neurological deficits. Also, the Alzet pump was removed at the end of each experiment, and the amount of the drug remaining in the pump was checked. If there was still residual drug in the pump, the data from that rat were excluded from the final analysis.

### Statistical analysis

All results are presented as mean ± SEM. Statistical analyses were performed by Student’s t test, one-way ANOVA followed by Dunnett’s multiple-comparison tests, or two-way repeated-measures ANOVA followed by Bonferroni’s multiple-comparison tests where appropriate. A value of *P*<0.05 was considered statistically significant.

## Results

### Nocifensive behavior to mechanical or heat stimulation of the tongue

**Figure 1:**
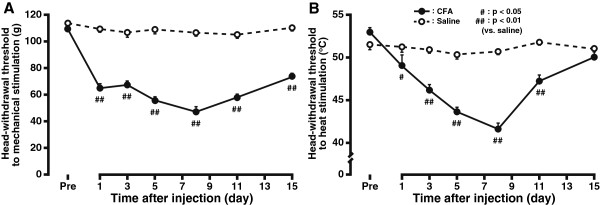
**Persistent behavioral hypersensitivity induced by local injection of CFA emulsion into the tongue.** Shown are the time course of changes in mechanical head withdrawal threshold (MHWT, **A**) and heat head withdrawal threshold (HHWT, **B**) in both saline- and CFA-treated groups. Compared with the saline control, CFA injection into the tongue elicited a significant reduction of MHWT and HHWT, with a peak reached at 8 day after treatment. Pre: baseline. *n* = 7 in each group. Error bars indicate SEM.

Clear signs of local tissue inflammation with remarkable redness and swelling of the injected site could be observed after submucosal injection of 5 μL complete Freund’s adjuvant (CFA) into the tongue, which lasted for at least 2 weeks (data not shown). A significant reduction of mechanical head withdrawal threshold (MHWT) was observed on the side ipsilateral to CFA injection as early as day 1 after CFA injection (day 1 after CFA injection *vs*. baseline, 65.7 ± 2.8 g *vs*. 109.7 ± 0.7 g; *n* = 7, *P*<0.01, Figure 1A). Then, the MHWT continued to decrease and reached the peak on day 8 (47.6 ± 3.6 g, *P*<0.01). Noticeably, this CFA-induced mechanical allodynia maintained until the end of the experimental period (day 15 after CFA injection, 73.4 ± 2.0 g, *P*<0.01, Figure 1A).

CFA injection into the tongue also resulted in a significant decrease in heat head withdrawal threshold (HHWT) (Figure 1B). HHWT was reduced on day 1 after CFA injection, peaked on day 8 (41.6 ± 0.7°C, *n* = 7, *P*<0.01) but gradually returned to the baseline level on day 15 (50.1 ± 0.9°C, *P*>0.05). Injection of the vehicle (0.9% isotonic saline, *n* = 7) into the tongue did not produce any obvious alteration in either MHWT or HHWT in any time points (Figure 1). Both groups of animals showed normal gross behavior and weight gain during the experimental period (data not shown). These results suggest that CFA injection into the tongue could indeed cause pronounced mechanical and thermal hypersensitivity, thus establishing a novel behavioral model of inflammatory tongue pain in adult rodents.

### Tongue histology

**Figure 2:**
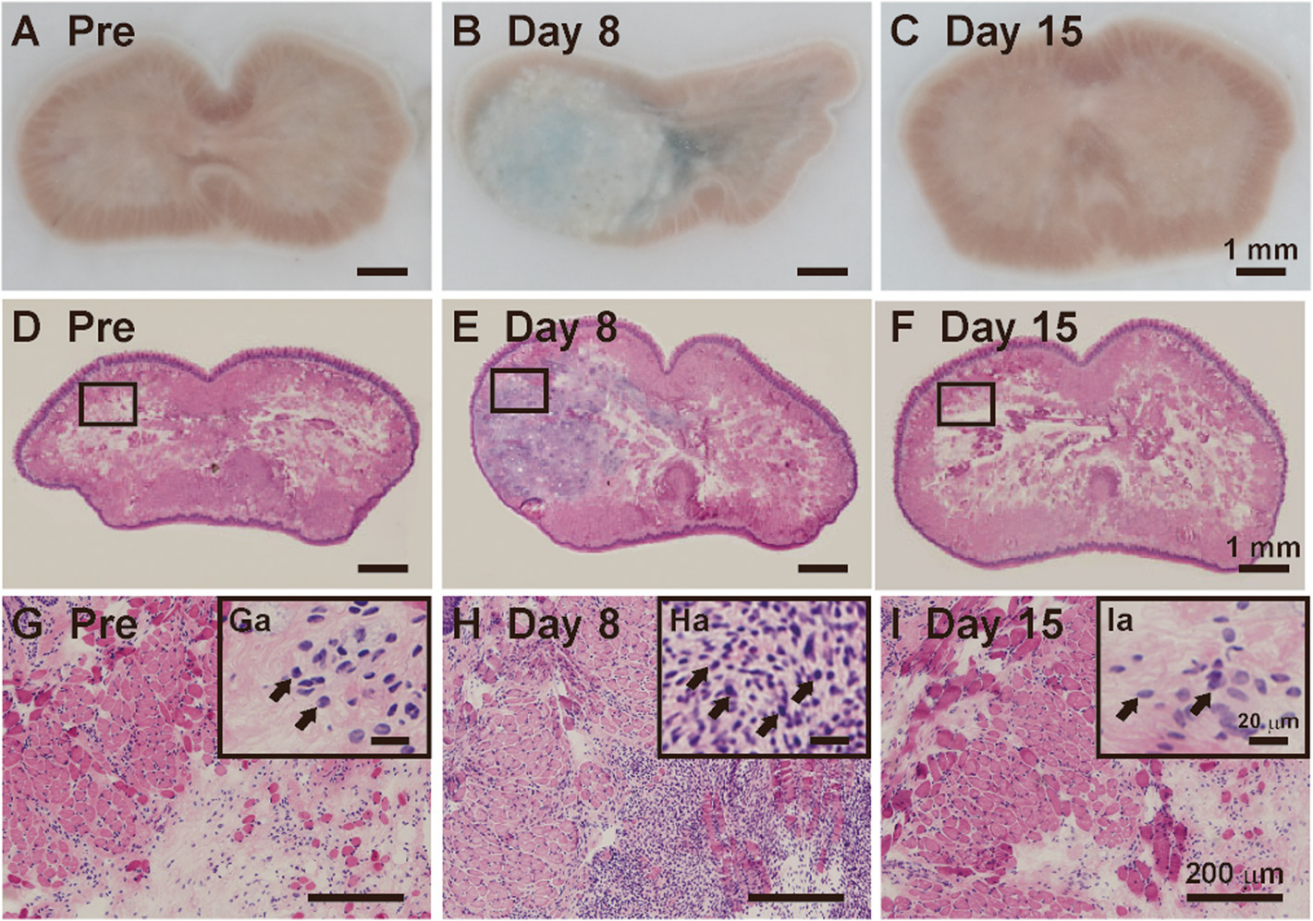
**Local inflammatory responses of the tongue after CFA injection.** Shown are the representative histological staining of the tongue before (**A**, **D**, **G**, and **Ga**), on day 8 (**B**, **E**, **H**, and **Ha**), and day 15 (**C**, **F**, **I**, and **Ia**) after CFA injection. **A** to **C**, Evans Blue staining; **D** to **I**, HE staining; **G** to **I** are enlarged photographs taken from the rectangle area in **D** to **F,** respectively. CFA injection into the tongue resulted in dramatic plasma extravasation and inflammatory cell infiltration on day 8 but not on day 15. Arrows in Ga-Ia indicate lymphocytes infiltrated into the tongue. Scale bars: 1 mm in **A** to **F**, 200 μm in **G** to **I**, 20 μm in Ga to Ia.

To confirm the occurrence of inflammation in the tongue after CFA injection, hematoxylin and eosin (HE) staining and Evans Blue staining were performed on days 8 and 15 after local CFA injection (Figure 2). The Evans Blue experiment revealed severe signs of plasma extravasation in the CFA-injected tongue on day 8, but not on day 15 (Figure 2A, B, and C). In addition, HE staining demonstrated a dramatic tissue infiltration of inflammatory cells (for example, lymphocytes as indicated by arrows in Figure 2Ga-Ia) in the CFA-injected tongue on day 8 but not on day 15 (Figure 2D to I).

### ERK phosphorylation in Vc and C1-C2

**Figure 3:**
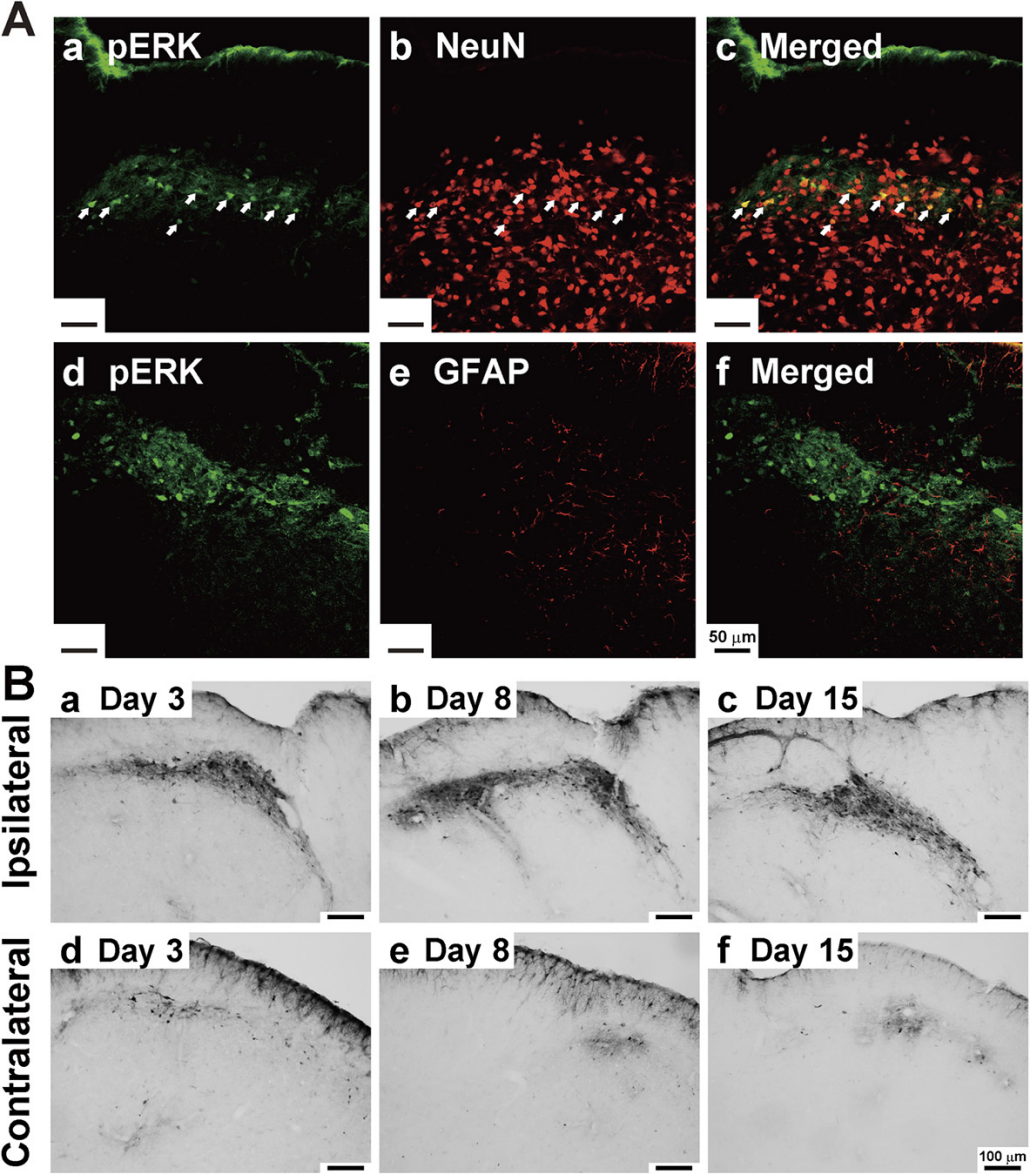
**Neuronal localization and bilateral distribution of pERK expression in the Vc and C1-C2.** (**A**) Photomicrographs of ERK, NeuN and GFAP staining in the Vc following noxious mechanical stimulation of the tongue on day 8 after CFA injection. pERK-IR was shown as green fluorescence (**Aa** and **Ad**). NeuN and GFAP were visualized with red fluorescence (**Ab** and **Ae**). Overlap of left and middle panels revealed double fluorescence (**Ac** and **Af**), which was only identified for p-ERK and NeuN staining, indicating the selective induction of pERK-IR in neurons. Arrows indicate the double-labeled cells. Scale bars: 50 μm. (**B**) pERK-IR cells following noxious mechanical stimulation of the tongue in ipsilateral (**Ba**, **Bb**, and **Bc**) and contralateral (**Bd**, **Be**, and **Bf**) Vc and C1-C2 on day 3 (**Ba** and **Bd**), day 8 (**Bb** and **Be**) and day 15 (**Bc** and **Bf**) after CFA injection. The expression of pERK-IR cells was detected in both sides of Vc and C1-C2 regions with an ipsilateral dominance. Most pERK-IR cells were located in the superficial layer and dorsomedial portion of Vc and C1-C2. Scale bars: 100 μm.

After verifying the new model of inflammatory tongue pain through behavioral and histological approaches, we next sought to elucidate possible activation (phosphorylation) of extracellular signal-regulated kinase (ERK) in the brainstem in response to CFA-evoked persistent inflammatory pain. To this end, we first performed double immunofluorescence staining to identify the nature of phosphorylated ERK (pERK) immunoreactive (IR) cells induced by noxious mechanical stimulation on day 8 after CFA injection into the tongue (Figure 3). Almost all pERK-IR cells were double stained with NeuN (Figure 3 Aa to Ac) but not glial fibrillary acidic protein (GFAP) (Figure 3 Ad to Af), indicating that CFA-evoked phosphorylation of ERK is restricted to neurons.

As shown in Figure 3B, a large number pERK-IR cells were expressed in both ipsilateral and contralateral trigeminal spinal subnucleus caudalis (Vc) and upper cervical spinal cord (C1-C2) after noxious mechanical stimulation of the tongue on day 3 (Figure 3 Ba and Bd), day 8 (Figure 3 Bb and Be), and day 15 (Figure 3 Bc and Bf) after CFA injection. The pERK-IR cells were substantially located in the dorsomedial portion of Vc and C1-C2 where the mandibular nerve terminates [10], and mainly segregated in the superficial layers (layer I/II), with a few scattered in the deep layer (layer III/IV).

**Figure 4:**
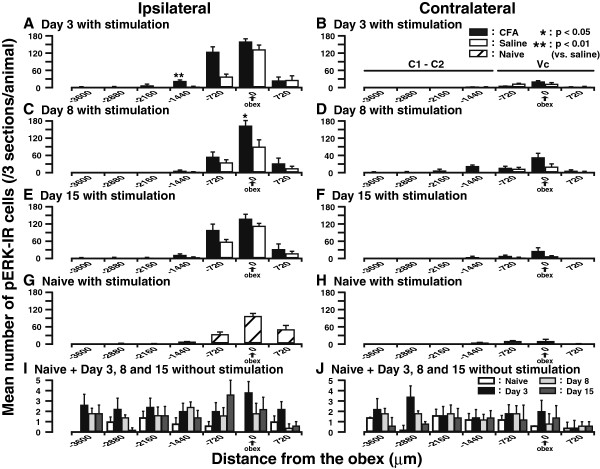
**Rostro-caudal arrangement of the mean number of pERK-IR cells evoked by noxious mechanical stimulation of the tongue through Vc and C1-C2 both ipsilateral (A, C, and E) and contralateral (B, D, and F) to saline and CFA injection.** (**A**) and (**B**), day 3; (**C**) and (**D**), day 8; (**E**) and (**F**), day 15 after saline and CFA injection into the tongue. Rostro-caudal arrangement of the mean number of pERK-IR cells following noxious mechanical stimulation of the tongue in ipsilateral (**G**) and contralateral (**H**) Vc and C1-C2 in naive rats. Rostro-caudal arrangement of the mean number of pERK-IR cells in ipsilateral (**I**) and contralateral (**J**) Vc and C1-C2 in naive and CFA-injected rats without noxious stimulation. The horizontal bars indicate the range of Vc and C1-C2 across the rostrocaudal dimension. *n* = 5 in each group. Error bars indicate SEM.

The rostrocaudal distribution of pERK-IR cells in the ipsilateral and contralateral Vc and C1-C2 following saline and CFA injection into the tongue is summarized in Figure 4. The largest number of pERK-IR cells was observed at the obex level on days 3 (Figure 4A and B), 8 (Figure 4C and D), and 15 (Figure 4E and F) after noxious mechanical stimulation in CFA- or saline-injected rats. The number of pERK-IR cells was significantly larger in CFA-injected rats compared with that of saline-injected rats at 1,440 μm caudal from obex on day 3 (*n* = 5, *P*<0.01) and at obex on day 8 (*P*< 0.05) for the ipsilateral side. The largest number of pERK-IR cells was also observed at the obex level after noxious mechanical stimulation in naive rats (Figure 4G and H). However, there were no significant differences in the number of pERK-IR cells between saline-injected and naive rats in each segment. Moreover, pERK-IR cells were observed in the ipsilateral (Figure 4I) and contralateral (Figure 4J) Vc and C1-C2 on days 3, 8, and 15 after CFA injection without noxious mechanical stimulation and also in naive rats. The ipsilateral side of Vc and C1-C2 (Figure 4A, C, E, and G) exhibited more p-ERK-IR cells than the contralateral side following noxious mechanical stimulation (Figure 4B, D, F, and H).

**Figure 5:**
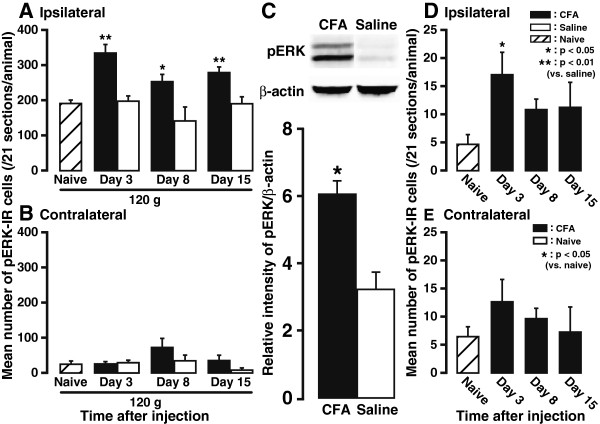
**Quantification of the time course of pERK expression in the Vc and C1-C2 after saline and CFA injection into the tongue.** Shown is the mean number of pERK-IR cells triggered by noxious mechanical stimulation of the tongue in ipsilateral (**A**) and contralateral (**B**) Vc and C1-C2 on days 3, 8, and 15 after saline and CFA treatment and in naive rats. (**C**) Normalized amount of pERK protein expression on day 8 after saline or CFA injection into the tongue. β-actin was used as a loading control. (**D** and **E**) Quantification of the time course of pERK expression in Vc and C1-C2 neurons after CFA injection into the tongue and in naive rats without noxious stimulation. Shown is the mean number of pERK-IR cells in ipsilateral (**D**) and contralateral (**E**) Vc and C1-C2 on days 3, 8, and 15 after CFA injection. CFA injection into the tongue initiated a much stronger pERK expression in Vc and C1-C2 than the control group, regardless of the presence of noxious stimulation. *n* = 5 in each group. Error bars indicate SEM.

The number of pERK-IR cells in ipsilateral Vc and C1-C2 was significantly larger in CFA-injected rats with noxious mechanical stimulation than that in saline-injected rats at each time point (*n* = 5, Figure 5A), though no group difference was detected in the number of pERK-IR cells in the contralateral side (Figure 5B). In addition, the number of pERK-IR cells in ipsilateral (but not contralateral) Vc and C1-C2 was still much larger in CFA-injected rats without noxious mechanical stimulation than that in naive rats at each time point (*n* = 5, Figure 5D and E). Consistently, Western blot data showed that pERK protein expression in ipsilateral Vc and C1-C2 was dramatically upregulated after CFA injection into the tongue compared with saline-injected group (*n* = 5, *P*<0.05, Figure 5C). Taken together, these data indicate that CFA-evoked inflammatory state resulted in a much stronger ERK activation in Vc and C1-C2 neurons in both presence and absence of noxious mechanical stimulation of the tongue. Furthermore, the layer and somatotopic arrangement of pERK-IR neurons are in line with the distribution of orally-innervated nociceptive neurons in Vc and C1-C2 as reported previously [10,12].

### Effects of PD98059 on nocifensive behavior and ERK phosphorylation

**Figure 6:**
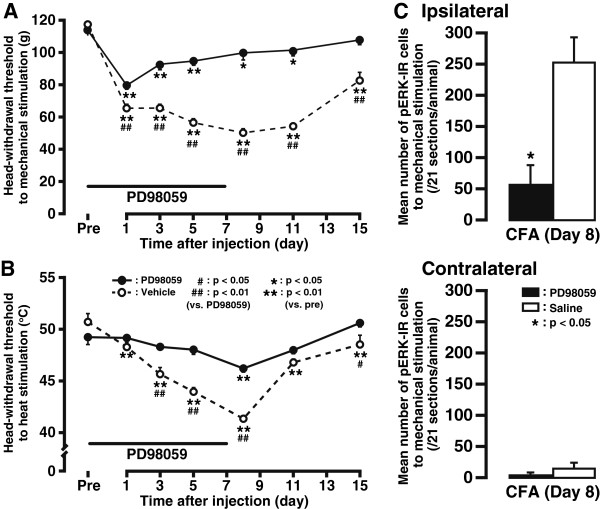
**Effects of continuous i.t. administration of the MEK inhibitor, PD98059, on the development of mechanical allodynia and heat hyperalgesia and CFA-induced ERK phosphorylation.** Time course of changes in MHWT (**A**) and HHWT (**B**) in both vehicle- and PD98059-treated groups. *n* = 5 to 6 in each group. (**C**) The mean number of noxious stimuli-evoked pERK-IR cells in the ipsilateral and contralateral Vc and C1-C2 on day 8 after CFA injection into the tongue with continuous i.t. administration of PD98059 or saline. *n* = 3 in each group. Error bars indicate SEM.

To investigate the contribution of ERK phosphorylation in Vc and C1-C2 neurons to the development of mechanical allodynia and heat hyperalgesia in the tongue, PD98059 (0.1 μg/μL) or vehicle (10% DMSO) was intrathecally applied to the CFA-treated rats for 7 days. Successive intrathecal (i.t.) administration of PD98059 effectively suppressed the decrement of MHWT compared with vehicle-administrated CFA-treated rats from days 1 to 15, however, MHWT was still significantly lower in PD98059-administrated CFA-treated rats from days 1 to 11 compared with the threshold value before PD98059 administration (Pre), suggesting a partial but not complete inhibition of mechanical allodynia by PD98059 administration (Figure 6A). Specifically, the mean peak value of MHWT recovered from 51.0 ± 1.7 g in vehicle control (*n* = 6) to 99.7 ± 4.5 g in PD98059-treated group (*n* = 5, *P*<0.01) on day 8 after CFA injection. In contrast, HHWT was not changed on days 1 to 15 (except on day 8 ) in PD98059-treated CFA-injected rats compared with the pre-drug baseline value, but was indeed higher than that of vehicle-administrated CFA-treated rats on days 3, 5, 8, and 15 (Figure 6B). The averaged peak HHWT in PD98059-treated group (46.2 ± 0.3°C, *n* = 6) was higher than that of the vehicle control (41.4 ± 0.3°C, *n* = 5, *P*<0.01). These results demonstrate that the blockade of ERK phosphorylation in Vc and C1-C2 neurons partially depressed the development of CFA-induced mechanical allodynia and heat hyperalgesia in the tongue. Lastly, i.t. administration of PD98059 also caused profound suppression of pERK expression in the ipsilateral Vc and C1-C2 on day 8 after CFA injection (PD98059 *vs*. vehicle, 254.3 ± 38.6 *vs*. 58.3 ± 29.9; *n* = 3, *P*<0.05), whereas no change in the number of pERK-IR cells was observed following i.t. administration of PD98059 in the side contralateral to the CFA injection (Figure 6C).

### Involvement of mGluR5 in ERK-mediated inflammatory pain signaling

**Figure 7:**
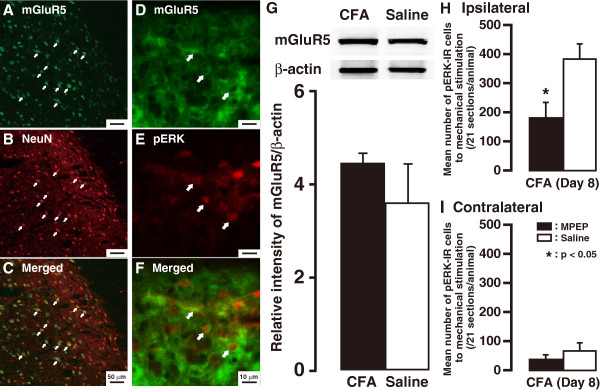
**Neuronal localization and expression of mGluR and effects of continuous i.t. administration of the mGluR5 antagonist, MPEP, on CFA-induced ERK phosphorylation.** Photomicrographs of mGluR5 (**A**) and NeuN (**B**) staining in the Vc following noxious mechanical stimulation of the tongue on day 8 after CFA injection. (**C**) Overlay of the double imunofluoresence labeling, showing the neuronal nature of mGluR5-IR cells. Arrows indicate the double-labeled cells. (**D**, **E**, **F**) Photomicrographs of mGluR5 (D) and pERK (E) staining in the Vc on day 8 after CFA injection. (F) Overlay of the double imunofluoresence labeling, suggesting the colocalization of mGluR5 with pERK (arrows). (G) Normalized amount of mGluR5 protein in Vc and C1-C2 on day 8 after saline or CFA injection into the tongue. β-actin was used as a loading control. The mean number of noxious stimuli-triggered pERK-IR cells in the ipsilateral (**H**) and contralateral (**I**) Vc and C1-C2 on day 8 after CFA injection into the tongue with continuous i.t. administration of MPEP or saline. MPEP administration effectively decreased CFA-induced pERK upregulation. n = 4 in each group. Error bars indicate SEM.

To evaluate the hypothesis that metabotropic glutamate receptor 5 (mGluR5) might act as an upstream activator/mediator in ERK-dependent inflammatory pain signaling, we next performed the double immunofluorescence labeling to identify the nature of mGluR5-IR cells following noxious mechanical stimulation of the tongue on day 8 after CFA injection. Most mGluR5-IR cells showed NeuN immunoreactivity in Vc and C1-C2 (Figure 7A, B, and C) and mGluR5 was expressed in a rim region of pERK-IR cell bodies (Figure 7D, E, and F), indicating that ERK was phosphorylated in neurons in which mGluR5 was expressed. On day 8 after CFA injection, mGluR5 protein expression in Vc and C1-C2 was not changed compared with saline-treated group (Figure 7G).

**Figure 8:**
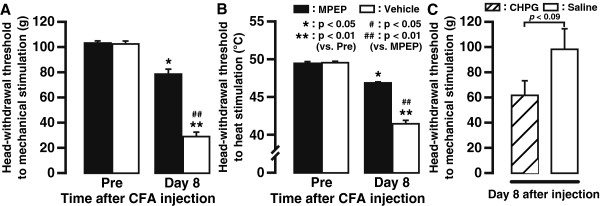
**Effects of i.t. infusion of MPEP or vehicle on MHWT (A) and HHWT (B) values measured on day 8 after CFA injection into the tongue.** Successive i.t. MPEP treatment partially but significantly diminished CFA-evoked behavioral hypersensitivity. (**C**) Effects of i.t. 7 days successive administration of CHPG or saline on MHWT values measured on day 8. *n* = 5 in each group. Error bars indicate SEM.

Successive 2-methyl-6-(phenylethynyl)-pyridine (MPEP) administration yielded a marked decrease in the number of pERK-IR cells in the ipsilateral Vc and C1-C2 (MPEP *vs*. vehicle, 183.7 ± 50.2 *vs*. 385.9 ± 49.0; *n* = 4, *P*<0.05) on day 8 after CFA injection into the tongue (Figure 7H), whereas no change was found in the contralateral side (Figure 7I). Continuous i.t. administration of MPEP for 7 days partially but significantly suppressed the decrement of both MHWT (MPEP *vs*. vehicle, 89.0 ± 2.9 g *vs*. 31.8 ± 2.5 g; *n* = 5, *P*<0.01) and HHWT (MPEP *vs*. vehicle, 46.9 ± 0.1°C *vs*. 41.5 ± 0.4°C; *n* = 5, *P*<0.01) on day 8 after CFA injection (Figure 8A and B). However, both MHWT and HHWT values were still lower in MPEP-administrated CFA-treated rats than the threshold value before CFA injection (Pre) on day 8 (*P*<0.05), implicating an incomplete abolishment of CFA-induced mechanical and thermal hypersensitivity by MPEP administration. Continuous i.t. administration of (RS)-2-Chloro-5-hydroxyphenylglycine (CHPG) for 7 days induced the decrement of MHWT on day 8 compared with saline-administrated group in naive rats (CHPG *vs*. saline, 62.3 ± 10.9 g *vs*. 98.9 ± 15.5 g; *n* = 5, *P*<0.09), implicating that mGluR5 activation in Vc and C1-C2 neurons is sufficient to induce mechanical allodynia in the tongue (Figure 8C).

## Discussion

### Inflammatory tongue pain model

In spite of previous extensive research efforts into the pathogenesis of extra-oral pain, limited information is now available concerning pathological pain occurring in the tongue, which causes great difficulty in diagnosis and treatment of tongue-related diseases (for example, burning mouth syndrome) in the clinic [44,45] . Therefore, it is quite necessary to devise new preclinical models of pathological tongue pain in order to gain better understanding of the underlying mechanisms and hence provide more effective therapeutic approaches. In this context, several models of orofacial neuropathic pain induced by lingual nerve injury (ligation, sectioning, constriction, and so on) have been previously developed and intensively characterized [46-50]. More recently, our lab also established a new animal model of lingual neuropathic pain by crushing the lingual nerve with an arterial clamp (30 g, 30 s), leading to marked mechanical allodynia and heat hyperalgesia in the tongue ipsilateral to the nerve injury [51]. However, to date, there are only very few papers reporting any models of inflammatory tongue pain by injecting capsaicin [12] or formalin [8] into the anterior part of the tongue. Moreover, in these studies, only spontaneous pain-related behaviors (for example, tongue protrusion, face scratching, or rubbing) were used to indirectly evaluate the tongue pain [8]. In contrast to the extra-oral structures, no standard methods are available to directly evaluate behavioral phenotypes of evoked pain hypersensitivity evolving in the tongue. Previously, behavioral and neuronal hypersensitivity have been observed in some orofacial inflammatory pain models following CFA injection into temporomandibular joint [1,52-54], masseter muscle [6,55,56], or facial skin [5,57]. Thus, in the present study, we sought for the first time to establish a novel model of inflammatory tongue pain by injecting 5 μL CFA emulsion into the anterior dorsolateral two-thirds of the tongue in adult rats.

Our results show that CFA injection into the tongue elicited clear signs of local tissue inflammation concomitant with significant reductions in MHWT and HHWT. The results directly indicate the occurrence of mechanical and heat hypersensitivity in the inflamed tongue in rats. Interestingly, the reduction of HHWT, but not MHWT, recovered on day 15 following CFA injection, although the existence of thermal hyperalgesia and mechanical allodyniafollowing CFA injection into the upper lip/whisker pad continued for a few weeks [58]. It is well known that the transient receptor potential vanilloid 1 (TRPV1), activated by noxious heat (>43°C) stimuli, is dominantly expressed in small-sized primary afferent neurons and functions to modulate inflammation and pain [59-61]. Indeed, CFA-induced heat hyperalgesia depends on functional changes in TRPV1 [62-64]. On the other hand, one previous retrograde labeling study showed that cell bodies of trigeminal ganglion neurons innervating the lip are significantly smaller in size than those innervating the tongue [8]. Therefore, it is reasonable to speculate that the tongue is innervated by relatively fewer numbers of small-sized TRPV1-expressing trigeminal ganglion neurons, which may cause the different time course between MHWT and HHWT measurements following CFA injection into the tongue.

### Roles of pERK in Vc and C1-C2 neurons in CFA-induced behavioral hypersensitivity

ERK is a member of the MAPK family that can be phosphorylated after various types of noxious stimuli [65-67]. In the trigeminal system, ERK was found to be phosphorylated in many Vc and C1-C2 neurons within 5 min after capsaicin injection into various orofacial regions, and the number of pERK-IR cells increased following increases in the stimulus intensity [12,20,21,24]. These findings strongly suggest that ERK phosphorylation in Vc and C1-C2 neurons is a reliable marker of excitable neurons following noxious stimulation of the orofacial region. In the current study, we demonstrated that ERK phosphorylation substantially occurred in Vc and C1-C2 neurons following noxious pinch stimulation of the tongue, and pERK-IR neurons were mostly located in laminae I and II, which is in line with the distribution of nociceptive neurons in this area. Here, it is important to note that expression of pERK-IR in Vc and C1-C2 exhibited an ipsilateral dominance, although mechanical stimulation of the tongue could also induce some pERK-IR cells in the contralateral side. First, this bilateral distribution of pERK-IR cells is in accordance with previous results showing bilateral terminations of mandibular afferents in the brainstem [68]. Second, our previous work found that injection of capsaicin into the tongue caused a similar bilateral activation of ERK in the Vc, consistent with the present data [12]. Third, the ipsilateral dominance of pERK expression may be related to specific injections site of the tongue according to one previous report [69].

The pERK-IR cell expression following capsaicin injection into the whisker pad skin or masseter muscle was restricted within a rostrocaudally narrow area in the Vc and C1-C2. However, the distribution of pERK-IR cells was more widespread along the rostrocaudal dimension after capsaicin injection into the digastric and sternohyoideus muscle [21]. In this study, the largest number of pERK-IR cells was aggregated at the obex level and distributed within a quite narrow area (-720 μm to 720 μm from obex). This rostrocaudal arrangement of pERK-IR cells is in agreement with the observations on ERK activation in response to capsaicin injection into the tongue [12]. Furthermore, the number of pERK-IR cells and the amount of pERK protein expression in ipsilateral Vc and C1-C2 were significantly larger in CFA-injected rats than those in saline-injected rats regardless of the presence of noxious stimulation. Also, most pERK-IR cells were located in the dorsomedial portion of the Vc, where the third branch of the trigeminal nerve (mandibular nerve) terminates [10]. Additionally, there were no significant differences in the number of pERK-IR cells induced by noxious stimulation to the tongue between saline-injected and naive rats in each segment, indicating that the ERK activation at the obex level caused by saline injection is most likely due to the noxious mechanical stimulation. Taken together, these findings indicate that noxious primary afferents from the tongue predominantly project to a narrow area around the obex in the Vc with somatotopic organization. Furthermore, CFA injection into the tongue modified the excitability of nociceptive neurons in the Vc and C1-C2 by enhancing the activation of ERK irrespective of the pre-exposure to any noxious stimulus.

Activated forms of ERK could produce exaggerated pain sensations under both inflammatory and neuropathic conditions [15,70-72]. In this respect, we found that the number of pERK-IR cells in ipsilateral Vc and C1-C2 was significantly larger in CFA-injected rats than saline-injected control. Moreover, successive i.t. administration of the MEK1/2 inhibitor PD98059 effectively attenuated the mechanical and heat hypersensitivity in CFA-injected rats, accompanied with an apparent decrease in the number of pERK-IR neurons in the ipsilateral Vc. These observations suggest that the ERK pathway is involved in the enhancement of the excitability of Vc and C1-C2 neurons following CFA injection into the tongue, resulting in the initiation and/or maintenance of mechanical and heat hypersensitivity in the inflamed tongue.

### Involvement of mGluR5 in ERK-mediated inflammatory tongue pain

The group I mGluRs (mGluR5 and mGluR1) are thought to play vital roles in mediation or modulation of nociceptive transmission at different levels of the central nerve system [73-76]. mGluR5 mRNA and its protein are shown to be distributed in the spinal trigeminal nucleus [77,78]. mGluR5 contributes to the induction of long-term potentiation of primary afferent synaptic transmission in the superficial layer of the Vc [33], and modulates inflammatory nociceptive plasticity via downstream activation of ERK signaling in the spinal cord [34-36]. The amount of glutamate which is an endogenous ligand of mGluR5 increases in the dorsal horn following peripheral injection of inflammatory agents [79]. All of these previous findings make it very likely that mGluR5 serves as an upstream activator for CFA-induced ERK phosphorylation. Supporting this hypothesis, we documented that mGluR5 was colocalized with pERK in Vc and C1-C2 neurons. Moreover, continuous i.t. administration of the selective mGluR5 antagonist MPEP significantly blocked CFA-evoked mechanical and heat hypersensitivity in the inflamed tongue and reduced the number of pERK-IR cells in ipsilateral Vc and C1-C2. On the other hand, direct mGluR5 activation by i.t. CHPG administration induced mechanical allodynia in the tongue in naive rats. The present findings suggest that the mGluR5 activation in Vc and C1-C2 neurons by CFA-evoked glutamate release in the dorsal horn mediates ERK phosphorylation, and then Vc and C1-C2 neuronal excitability is enhanced, leading to mechanical and heat hypersensitivity in the rats with CFA injection into the tongue.

However, pre-treatment with i.t. administration of MPEP failed to completely abolish the upregulation of pERK-IR cells and the reduction of MHWT and HHWT on day 8 after CFA injection into the tongue, indicating that there are other upstream activators for neuronal hyperexcitability in Vc and C1-C2. For instance, ionotropic glutamate receptors and some other protein kinases play an important role in ERK-mediated enhancement of neuronal excitability in the dorsal horn [11,66,80]. In addition, inflammation-evoked ERK activation, which is required for nociceptive plasticity, is also the downstream of mGluR1 [36]. Antisense oligonucleotide knockdown of spinal mGluR1 attenuates heat hyperalgesia and mechanical allodynia in rats with CFA injection into the hindpaw [81]. Future studies are thus warranted to explore these possibilities and to dissect the candidate downstream effectors of ERK signaling responsible for the CFA-induced inflammatory nociceptive hypersensitivity in the tongue.

## Conclusions

The present study established a novel animal model of inflammatory tongue pain in rats, and explored potential roles of the mGluR5-ERK signaling in the development of mechanical and heat hypersensitivity that evolved in the inflamed tongue. Further elucidation of the mechanisms underlying this model of pathological tongue pain may shed new light on the pathogenesis and therapeutic strategies of some clinically relevant, tongue-associated diseases such as burning mouth syndrome.

## Abbreviations

BSA: Bovine serum albumin; C1-C2: Upper cervical spinal cord; CFA: Complete Freund’s adjuvant; CHPG: (RS)-2-Chloro-5-hydroxyphenylglycine; DMSO: Dimethyl sulfoxide; EMG: Electromyographic; GFAP: Glial fibrillary acidic protein; HE: Hematoxylin and eosin; HHWT: Heat head withdrawal threshold; IR: Immunoreactive; i.t.: Intrathecal; MAPK: Mitogen-activated protein kinase; mGluR5: Metabotropic glutamate receptor 5; MHWT: Mechanical head withdrawal threshold; MPEP: 2-methyl-6-(phenylethynyl)-pyridine; NGS: Normal goat serum; PBS: Phosphate-buffered saline; pERK: Phosphorylated extracellular signal-regulated kinase; RT: Room temperature; TRPV1: Transient receptor potential vanilloid 1; TBST: Tris-buffer saline containing 0.1% Tween 20; Vc: Trigeminal spinal subnucleus caudalis.

## Competing interests

All authors declare that they have no competing interests.

## Authors’ contributions

MGL and MS participated in the design of the experiments, performed the animal studies, analyzed the data and wrote the manuscript. SM executed the immunohistochemical analyses and revised the manuscript. KH participated in the design of the experiments and revised the manuscript. IS, KS, MK, and KU performed the immunohistochemical and Western blotting experiments. TT, AK, KO, and AF participated in the behavioral studies. KI conceived and designed the study, analyzed the data, and wrote the manuscript. All authors read and approved the final version of the manuscript.
